# The Clinical and Molecular Landscape of Rosette-Forming Glioneuronal Tumors

**DOI:** 10.3390/biomedicines12102325

**Published:** 2024-10-12

**Authors:** Zijiang Yang, Xiaobiao Zhang

**Affiliations:** 1 Department of Neurosurgery, Zhongshan Hospital, Fudan University, Shanghai 200030, China; 22111210107@m.fudan.edu.cn; 2 Cancer Center, Zhongshan Hospital, Fudan University, Shanghai 200030, China; 3 Digital Medical Research Center, Fudan University, Shanghai 200030, China

**Keywords:** rosette-forming glioneuronal tumors, molecular pathogenesis, immunohistochemistry, diagnostic challenges, therapeutic strategies

## Abstract

Background: Rosette-Forming Glioneuronal Tumors (RGNTs) are rare, typically benign central nervous system tumors primarily located in the fourth ventricle and pineal region. Despite being classified as WHO grade I with generally favorable prognoses, RGNTs present complexities in their molecular mechanisms, occasional malignant transformation, and epidemiological characteristics that require further investigation. Method: This study systematically reviews the existing literature to analyze the epidemiological patterns, MRI characteristics, pathological features, diagnostic challenges, and molecular mechanisms associated with RGNTs, aiming to provide a comprehensive theoretical foundation for clinical practice and future research. Results: Through an in-depth review of recent studies, key molecular mechanisms, including mutations in *FGFR1*, *PIK3CA*, *TERT*, and *IDH1/2*, are highlighted. Additionally, the challenges in accurate diagnosis and the potential for misdiagnosis are discussed, emphasizing the importance of thorough molecular analysis in clinical settings. The literature indicates that RGNTs predominantly affect young adults and adolescents, with a slight female predominance. MRI typically reveals mixed cystic–solid lesions, often accompanied by hydrocephalus. Pathologically, RGNTs are characterized by a combination of neuronal and glial components, with immunohistochemical staining showing positivity for Synaptophysin and GFAP. High frequencies of *FGFR1* and *PIK3CA* mutations underscore the significance of these pathways in RGNT pathogenesis and progression. Although RGNTs generally exhibit low malignancy, the *TERT* mutations identified in some cases suggest a risk of malignant transformation. Conclusions: This study concludes that while current treatment strategies focus on surgical resection, integrating molecular diagnostics and targeted therapies may be essential for managing recurrent or refractory RGNTs. Future research should explore the impact of various gene mutations on tumor behavior and their correlation with clinical outcomes, to optimize individualized therapeutic strategies and improve patient survival and quality of life.

## 1. Introduction

Rosette-Forming Glioneuronal Tumors (RGNTs) are rare and distinctive CNS tumors characterized by their relatively mild clinical course and unique histopathological features. RGNT was first described in detail by Komori et al. in 2002 [1], occurring predominantly in the fourth ventricle and pineal region, although they have also been reported in other CNS sites such as the midbrain and spinal cord. Typically, these tumors are classified as WHO grade I tumors, meaning that they usually have low malignancy and a better prognosis [2]. However, despite their classification as low malignancy, the underlying molecular mechanisms of RGNTs and their phenomenon of occasional malignant transformation still require further investigation.

In recent years, molecular biology studies have revealed the presence of multiple key gene mutations in RGNTs [3], particularly mutations in the *FGFR1* gene, which usually occur in its tyrosine kinase structural domain, leading to aberrant activation of the receptor and promoting cell proliferation and tumor formation through the RAS-MAPK signaling pathway. In addition, *PIK3CA* [4] and *TERT* [5] gene mutations have also been detected in some RGNT cases, and these mutations are closely associated with the biological behavior of the tumor and the potential risk of malignant transformation. Although the existing studies have provided a foundation for our understanding of the molecular mechanisms of RGNTs, there are still many unanswered questions, such as how these mutations affect the clinical manifestations and prognosis of the tumor. Therefore, further studies and reviews are necessary to gain a deeper understanding of the pathological mechanisms of RGNTs and to provide more precise guidance for future clinical diagnosis and treatment.

This study aimed to explore the epidemiologic features, MRI features, pathologic features, diagnosis, and differential diagnosis of RGNTs, as well as their molecular mechanisms through a systematic review of the existing literature, to provide a solid theoretical foundation for clinical practice and future research.

## 2. Epidemiological Characteristics

RGNTs are a rare central nervous system tumor, with their exact incidence still not fully determined. Current literature suggests that RGNTs predominantly affect adolescents and young adults, with an average age of onset of around 26 years, though cases have been reported in individuals ranging from 4 to 81 years old. The ratio of patients aged 18 and above to those under 18 is approximately 2.8:1, while the ratio of patients aged 26 and above to those younger than 26 is about 1:1. The age distribution of RGNTs shows that the proportion of pediatric patients is significantly higher than that of adults. However, there is currently no clear molecular mechanism to explain this age-related disparity. The majority of reported RGNT cases in the literature originate from China [3], the United States [6], France [7], and Germany [8]. Due to the rarity of RGNTs and incomplete statistical data, this does not necessarily indicate a higher incidence of RGNTs in these regions.

Furthermore, there is a slight female predominance, with a female-to-male ratio of approximately 1.4:1 [3,9,10,11,12,13,14]. Although no definitive molecular mechanisms have been identified to explain this phenomenon, other central nervous system tumors, such as astrocytomas, also demonstrate a similar gender bias [15]. Future research may uncover the potential influence of gender-related factors on the development of these tumors.

RGNTs are most frequently found in the posterior fossa, particularly in the fourth ventricle and pineal region. Tumors located in the infratentorial region are significantly more common than those in the supratentorial region, with a ratio of approximately 3.6:1 [3,16]. RGNTs are commonly found in midline structures, such as the fourth ventricle and pineal region, which may be related to the neurodevelopmental origins of these tumors. However, no specific literature to date has reported a direct correlation between the midline predilection of RGNTs and their particular molecular mechanisms. During early neurodevelopment, the midline regions, including the fourth ventricle and pineal area, harbor neural progenitor cells, which serve as essential sources for both neurons and glial cells. Given the high proliferative capacity of these progenitor cells [17], it is hypothesized that they may be more susceptible to certain genetic mutations or signaling pathway aberrations, potentially leading to tumor formation. Although research on RGNTs is limited, some midline tumors, such as midline glioblastomas, are associated with mutations in genes involved in neurodevelopment (such as TP53, NF1, ATRX, PI3K, IDH1, and H3.3) [18,19,20]. These gene mutations may disrupt the normal development of neural progenitor cells in midline regions and promote tumorigenesis. RGNTS may share similar molecular mechanisms with these tumors. Existing studies indicate that RGNTs exhibit molecular characteristics such as FGFR1 gene mutations and PI3K/AKT pathway abnormalities [3]. Although these mechanisms may not be directly linked to the midline predilection, they could play a crucial role in the proliferation of neurons and glial cells in midline regions. Therefore, these molecular features may partially explain the formation of tumors in midline areas. Lastly, the unique microenvironment of midline regions, particularly around the ventricles and the pineal region, may support the growth and survival of tumor cells. These areas are rich in cerebrospinal fluid and possess distinct signaling environments, which may facilitate molecular abnormalities such as dysregulated signaling pathways or abnormal cell proliferation, thereby promoting tumor formation in midline regions. In the study by Duan et al. [12], five RGNT cases were included, with three cases located in midline regions and two in non-midline regions. The authors compared genetic alterations between RGNTs, dysembryoplastic neuroepithelial tumors (DNETs), and mixed glioneuronal tumors (MGNTs), although there has been limited research on the molecular differences between midline and non-midline RGNTs. Currently, the specific molecular mechanisms driving this midline predilection remain underexplored.

Additionally, a few cases have been reported in the spinal cord and midbrain tegmentum, and there are rare instances of multifocal or metastatic RGNTs [3,5,13,21,22]. Around 60% of patients present with symptoms related to hydrocephalus, such as headaches, nausea, vomiting, and gait instability [3,9].

Currently, gross total resection is considered the most effective treatment option [23]. Survival data indicate that the prognosis for RGNTs is generally favorable, especially in cases where the tumor is completely resected. The overall survival (OS) rate at 2 years post-diagnosis remains at 100% [9,10]. The 1.5-year disease-free survival rate is close to 100%, though the 10-year disease-free survival rate decreases to around 50% [9,10]. Due to its relatively benign pathological characteristics, the recurrence rate is low. In the study by Orestes et al., only 1 out of 35 patients experienced recurrence [6], suggesting that a subset of patients may face a risk of recurrence during long-term follow-up. Compared to other gliomas, the recurrence rate of RGNTs is lower, but further research is needed to address the treatment of recurrent cases.

In terms of tumor size, most RGNTs are less than or equal to 3.5 cm in diameter, with cases involving smaller tumors being approximately 3.2 times more common than those with tumors larger than 3.5 cm [9,16]. RGNTs are usually slow-growing, and patients are often diagnosed years after the onset of symptoms [24].

## 3. Neuroradiological Features

The most common sites of occurrence for RGNTs are the fourth ventricle and pineal region, though there are a few reported cases from other CNS regions, such as the midbrain tegmentum and spinal cord [5,21].

### 3.1. CT Features

On CT imaging [3,25,26]: the solid component of RGNTs typically appears to be hypo dense, while the cystic component demonstrates an even lower density, often presenting as a mixed solid-cystic structure. In some cases, CT scans may reveal intratumoral hemorrhage. These hemorrhagic foci are seen as hyperdense spots, contrasting with the hypodense solid tumor tissue. Hemorrhage is a relatively common but easily overlooked feature of RGNTs, particularly in early-stage CT scans, where it may be less apparent. In contrast-enhanced CT scans, RGNTs often show minimal or no enhancement, especially in the cystic portion of the tumor, which generally does not enhance. However, the solid component may exhibit peripheral or patchy enhancement, depending on the tumor’s specific tissue composition and vascular supply. Calcification is not a common feature, but it has been reported in a few cases and should be considered as a notable radiologic characteristic during diagnosis. Additionally, RGNTs are typically located in the midline posterior fossa, particularly in the fourth ventricle. In this location, the tumor may present as a hypodense mass effect and could be associated with CT findings indicative of hydrocephalus due to obstruction of cerebrospinal fluid flow.

### 3.2. MRI Features

MRI is the primary imaging modality for diagnosing RGNTs and typically shows the tumor as a cystic–solid mixed lesion. The morphology and anatomical location of the tumor significantly influence the clinical symptoms and imaging features observed in patients [10,23].

#### 3.2.1. T1-Weighted Imaging (T1WI)

On T1WI, RGNTs generally appear as a lesion with low to isointense signals. The cystic portions, due to their high fluid content, exhibit distinctly low signals, whereas the solid portions may present as isointense or slightly hypointense. These low signals reflect the differences in fluid content and tissue density within the tumor, aiding in its differentiation from other CNS tumors [10,27,28].

#### 3.2.2. T2-Weighted Imaging (T2WI)

On T2WI, RGNTs typically show a high signal, particularly in cystic regions, which exhibit high signals due to fluid accumulation. The signal intensity of the solid portion depends on the tumor’s histological composition and may vary between high and isointense. The high signal on T2WI helps to delineate the overall morphology of the tumor, providing crucial information for determining its boundaries and internal structure [10,27,28,29].

#### 3.2.3. FLAIR Sequence

FLAIR sequences are effective for further evaluating both the solid and cystic components of RGNTs by suppressing the cerebrospinal fluid signal. In FLAIR images, the cystic portions generally show low signals, while the solid portions may display high signals. This imaging modality clarifies the tumor’s boundaries, making it particularly useful for identifying the distribution and expansion of the solid components [27,28].

#### 3.2.4. Enhanced T1-Weighted Imaging (Enhanced T1WI)

In enhanced T1WI, the solid portion of RGNTs usually demonstrates mild to moderate enhancement, whereas the cystic portions, lacking significant blood supply, typically show no enhancement. The enhancement pattern may be patchy or unevenly distributed, reflecting angiogenesis within the tumor. Enhanced imaging is crucial for assessing the tumor’s vascular characteristics, confirming tumor boundaries, and determining its relationship with surrounding tissues. Additionally, enhanced T1-weighted imaging (T1WI) can be used to detect cerebrospinal fluid (CSF) dissemination in RGNTs, although such dissemination is uncommon. Tumor cells that have disseminated through the CSF typically form diffuse deposits along the surface of the spinal cord. These deposits appear as abnormal signals on the surface of the spinal cord and within the subarachnoid space, contrasting with the surrounding tissues, indicating that tumor cells have spread to these regions [3,10,16,26,27,28,30].

#### 3.2.5. Diffusion-Weighted Imaging (DWI) and Apparent Diffusion Coefficient (ADC) Maps

On DWI, RGNTs usually appear as a lesion with a low signal, while on ADC maps, it appears with a high signal intensity. This suggests a lower degree of diffusion restriction within the tumor, indicating a lower cell density. These features align with RGNTs’ typically low malignancy profile and help distinguish them from more aggressive tumors [26,31,32].

#### 3.2.6. FIESTA Sequence

The FIESTA (Fast Imaging Employing Steady-state Acquisition) sequence provides high tissue contrast and detailed clarity when evaluating RGNTs. The FIESTA sequence is particularly sensitive to fluid–tissue interfaces, where the cystic portions of RGNTs generally exhibit high signals, while the solid portions might show moderate signals. This sequence clearly delineates the tumor borders and internal structures, especially the transition areas between cystic and solid components. Additionally, FIESTA is highly sensitive to blood at rest or with a low flow velocity, enabling visualization of the tumor’s microvascular structures. This is particularly valuable for assessing the tumor’s blood supply and its relationship with adjacent neural tissues. Given RGNTs’ common location in the fourth ventricle and pineal region, tumor growth may obstruct cerebrospinal fluid circulation, leading to obstructive hydrocephalus. The FIESTA sequence excels in showing the tumor’s relationship to the cerebrospinal fluid space and in evaluating its compression and displacement of surrounding structures. Franzini et al. [24] described the performance of patients with recurrent RGNTs on MRI, further exploring the use of Gamma Knife radiotherapy in controlling tumor growth, and showed that MRI has an important role in monitoring tumor progression and response to treatment [33].

#### 3.2.7. Susceptibility-Weighted Imaging (SWI) Characteristics

In SWI sequences, RGNTs typically demonstrate intratumoral microhemorrhages. These appear as hypointense signals (low signal intensity), forming small dark areas within the tumor on the SWI images. Additionally, SWI can reveal potential calcifications within RGNTs. Similar to microhemorrhages, calcifications also present as hypointense regions, contrasting with the surrounding normal brain tissue. Although calcifications are not a common feature in RGNTs, when present, SWI is more sensitive than conventional T1- or T2-weighted imaging for detecting these areas. The high sensitivity of SWI allows for the detection of smaller and earlier-stage hemorrhages compared to standard MRI sequences. Moreover, SWI can reveal the heterogeneity of the tumor. In SWI images of RGNTs, different regions may exhibit varying degrees of hypointensity, reflecting the presence of multiple pathological processes within the tumor, such as hemorrhage, calcification, or other magnetic susceptibility effects. This heterogeneous hypointensity aids in identifying the various components of the tumor, providing further diagnostic insights [26].

#### 3.2.8. Magnetic Resonance Spectroscopy (MRS) Features of RGNTs

In some cases, MRS imaging has been performed on RGNT patients, revealing an elevated choline/creatine (Cho/Cr) and choline/N-acetylaspartate (Cho/NAA) ratio. The increase in choline levels is typically associated with heightened membrane turnover and metabolic activity, suggesting active cellular proliferation within the tumor. Additionally, in some patients, a lipid–lactate peak has been observed, indicating the presence of hypoxic or necrotic areas within the tumor. This finding is commonly associated with more aggressive tumor behavior and is often seen in malignant tumors, further underscoring the importance of monitoring such metabolic markers in RGNT diagnosis and characterization [3,26].

## 4. Pathological Features

**Figure 1:**
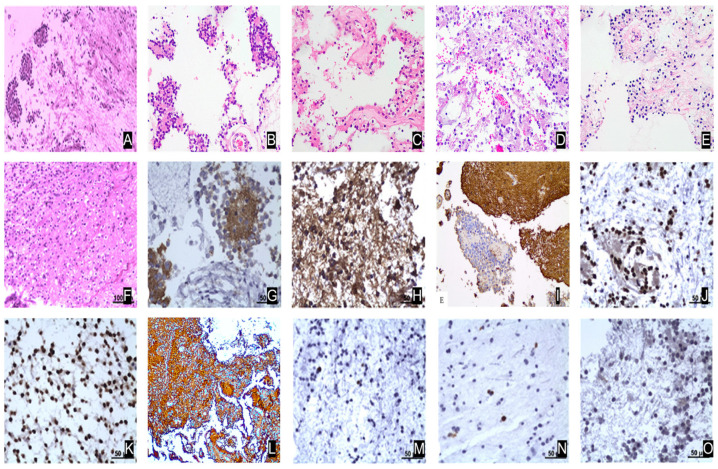
The histopathological and immunohistochemical features of rosette-forming glioneuronal tumors (RGNTs). (**A**) HE staining illustrates the characteristic biphasic structure of RGNTs, comprising distinct glial and neurocytic components [3]. (**B**–**E**) The neurocytic component is defined by rosette formations, where cells with sparse cytoplasm and densely packed nuclei surround eosinophilic neuropil cores or form perivascular pseudorosettes, as visualized with HE staining [10]. (**F**) The glial component consists of astrocytic cells, either spindle-shaped or stellate, forming a dense fibrillar matrix, which is reminiscent of pilocytic astrocytoma [3]. (**G**) Immunohistochemical staining for Synaptophysin reveals intense reactivity within the neuropil cores of neurocytic rosettes, emphasizing their neuronal differentiation [3]. (**H**) GFAP staining shows strong immunoreactivity in the glial background [3]. (**I**) The neurocytic regions show no GFAP expression, whereas the glial areas exhibit strong GFAP positivity [21]. (**J**) Olig-2 immunostaining shows reactivity in both neurocytic rosettes and (**K**) the glial background, suggesting that both components are derived from Olig2-expressing progenitor cells [3]. (**L**) S-100 protein expression is observed in the glial component but is absent from the rosettes and pseudorosettes, further distinguishing these components [10]. (**M**,**N**) Ki-67 labeling indicates low proliferation rates in both the neurocytic and glial elements, consistent with the typically indolent behavior of RGNTs [3]. (**O**) NeuN staining reveals focal reactivity in the neurocytic regions, confirming the neuronal identity of these structures [3].

RGNTs represent a rare subset of CNS tumors, with their pathological characteristics playing a crucial role in diagnosis. The histological architecture of RGNTs is intricate and varied, typically comprising a mix of neuronal and glial components that exhibit distinct histomorphological features (Figure 1A) [3,10].

### 4.1. Histological Characteristics

#### 4.1.1. Neuronal Components

The neuronal component of RGNTs is frequently organized into rosette-like structures. These structures are composed of small, tightly packed neuron-like cells that encircle a central fibrous or acellular matrix. The rosette-like formations are a hallmark pathological feature of RGNTs and are readily identifiable through histological staining. The neuron-like cells typically have small, round, or oval nuclei, have minimal cytoplasm, and are basophilic, with minimal nuclear heterogeneity. These rosette-like formations are commonly observed in HE (Hematoxylin and Eosin) staining, where they display a characteristic rose-like arrangement (Figure 1B–E) [1,10,34].

#### 4.1.2. Glial Components

The glial component in RGNTs is also significant and often manifests as slender, spindle-shaped cells, resembling those seen in pilocytic astrocytomas. In HE staining, the glial component displays dense nuclei with uniform nuclear morphology and inconspicuous cytoplasm. These cells are typically tightly packed and arranged in bundles or tangles. Compared to the neuronal component, the nuclei of the glial component are generally more elongated or oval with less heterogeneity. Additionally, the transition zone between the neuronal and glial components is more clearly delineated, and distinct boundaries between different cell populations can be observed in tissue sections (Figure 1F) [10,14,34].

### 4.2. Immunohistochemical (IHC) Features

Immunohistochemical staining is critical in the pathological diagnosis of RGNTs, as it helps further elucidate the tumor’s tissue origin and cellular composition based on the expression patterns of specific markers.

#### 4.2.1. Synaptophysin Positive Staining

Synaptophysin, a neuronal marker, shows strong positive staining in the neuronal components of RGNTs. This positive staining for synaptophysin supports the neuronal origin of the rosette-like structures, making it a crucial marker for differentiating RGNTs from other types of glioneuronal tumors (Figure 1G) [3,4,14].

#### 4.2.2. GFAP Positive Staining

Glial fibrillary acidic protein (GFAP), a specific marker for glial cells, exhibits positive staining in the glial component of RGNTs. GFAP positivity is essential in confirming the presence of glial cells within the tumor, aiding in the distinction between glial and neuronal components (Figure 1H,I) [3,14,21,35].

#### 4.2.3. OLIG2 and S100 Positive Staining

OLIG2 and S100 proteins are additional markers commonly used to identify glial cell components. In RGNTs, OLIG2protein is expressed in both neuronal and glial cells (Figure 1J,K) [3], while S100 is typically positive in the glial component (Figure 1L) [10]. The broad expression of OLIG2 indicates the coexistence of neuroglial components within the tumor, a feature often seen in gliomas [3,7,14,35,36].

### 4.3. Ki-67 Proliferation Index

Ki-67 is a marker used to assess the proliferative activity of tumor cells. Approximately 70% of RGNTs demonstrate a low Ki-67 proliferation index, usually below 1.6%, reflecting their low malignancy and slow growth characteristics (Figure 1M,N) [3]. Clinically, a low Ki-67 index is indicative of a better prognosis, but in rare cases, an elevated Ki-67 index may suggest a potential risk for malignant transformation [9].

### 4.4. Other Histological and Immunohistochemical Markers

#### 4.4.1. NSE, NeuN and Neurofilament Staining

Neuron-specific enolase (NSE), neuronal nuclei antigen (NeuN), and Neurofilament are neuron-specific markers that frequently exhibit positive staining in the neuronal component of RGNTs. The expression of these markers further confirms the presence of neuronal elements within the tumor, particularly in rosette-like structures (Figure 1O) [3].

#### 4.4.2. p53 and IDH1 R132H Staining

Although mutations in p53 and IDH1 R132H are relatively rare in RGNTs, their immunohistochemical staining can aid in distinguishing RGNTs from other types of gliomas. Abnormal expression of p53 typically suggests a TP53 gene mutation, whereas IDH1 R132H mutations are often associated with certain low-grade gliomas. In RGNTs, the positive or negative staining results for these markers can help rule out other tumor types [6,37,38].

### 4.5. Histological Heterogeneity and Special Cases

Histological heterogeneity in RGNTs is evident across different cases and tumor locations. In RGNTs located in the spinal cord or midbrain tegmentum, tumors may present with more complex histological structures and may even exhibit higher cellular heterogeneity and more aggressive behavior [5,21]. RGNTs in the midbrain tegmentum, which show greater cellular heterogeneity, often carry mutations in the *FGFR1* gene, and are linked to increased tumor cell proliferation and invasiveness. In spinal cord RGNTs, although these tumors exhibit typical RGNTs features histologically—such as rosette-like structures and a mix of neuronal and glial components—molecular studies suggest that these tumors may possess unique genetic mutation profiles. The frequency and types of gene mutations in spinal cord RGNTs differ from those in typical CNS RGNTs, indicating that RGNTs from different anatomical sites may display significant heterogeneity in cell proliferation, metabolic pathways, and extracellular matrix remodeling. For RGNTs in these regions, it is crucial to consider how their unique molecular characteristics might impact diagnosis and treatment.

The pathological features of RGNTs highlight their complexity as glioneuronal tumors. Accurate pathological diagnosis relies on a combination of various histological and immunohistochemical markers, which help differentiate RGNTs from other CNS tumors and provide critical pathological insight for clinical management.

## 5. Molecular Mechanisms

### 5.1. FGFR1 Gene Mutation

Mutations in the *FGFR1* (fibroblast growth factor receptor 1) gene are one of the primary molecular characteristics of RGNTs, with a mutation rate from 8% to 100% [3,5,7,8,11,12,13,16,39]. These mutations typically occur in the tyrosine kinase domain of FGFR1, leading to abnormal receptor activation. This activation promotes excessive cell proliferation and tumor formation through the RAS-MAPK signaling pathway [7,8,11]. Specifically, *FGFR1* mutations are key drivers of tumor development and have been widely observed across different RGNT cases. For example, Handa et al. [5] found that the *FGFR1 K656E* mutation is particularly common in RGNTs located in the midbrain tectum. This mutation may be associated with the activation of aberrant signaling pathways, but there is currently no clear evidence to suggest that it directly leads to increased tumor aggressiveness. Additionally, Kitamura et al. found that *FGFR1* gene mutations were present in both glial and neuronal components in some cases. However, the *FGFR1 N546K* mutation was found only in the glial component, but not in the neuronal component in one exceptional case. This suggests that although the two components originate from the same cell clone, different genetic alterations may have occurred during tumor development [16]. This finding suggests that *FGFR1* mutations play a critical role not only in tumorigenesis but also in the clinical manifestations and prognosis of RGNTs. Consequently, molecularly targeted therapies against FGFR1 mutations may offer a promising direction for future RGNT treatment, particularly in cases with aggressive mutations. These observations further highlight that RGNTs in different anatomical locations may exhibit significant molecular heterogeneity, which should be considered in their diagnosis and treatment.

### 5.2. PIK3CA Gene Mutation

*PIK3CA* gene mutations represent another important molecular feature in RGNT [3,39]. The *PIK3CA* gene encodes phosphatidylinositol-3-kinase (PI3K), which plays a crucial role in the PI3K/AKT/mTOR signaling pathway—a pathway integral to cell growth, proliferation, and survival. Mutations in the *PIK3CA* gene with a mutation rate from 11% to 75% [3,4,8,11,13,14,16,39] typically result in increased PI3K enzyme activity, driving sustained tumor cell growth and proliferation while inhibiting apoptosis, thus conferring a growth advantage to the tumor cells. Molecular analyses have identified *PIK3CA* mutations in RGNTs, suggesting that these mutations promote tumor growth and survival by activating the PI3K/AKT signaling pathway [4,29]. This mutation enhances the sensitivity of tumor cells to external growth signals, further boosting cell proliferation and malignant potential by inhibiting apoptotic pathways. Additionally, Bidinotto et al. [7] explored the interaction between *PIK3CA* and *FGFR1* mutations, proposing that these mutations may synergistically drive tumor formation and progression. The coexistence of these mutations underscores the complexity of RGNT pathology, highlighting the need to consider these molecular characteristics in diagnosis and treatment. These findings not only deepen our understanding of RGNTs’ molecular mechanisms but also provide potential targets for future therapeutic strategies. By studying key gene mutations such as *PIK3CA* and *FGFR1*, more precise treatments could be developed to address these complex tumor traits.

### 5.3. TERT Gene Mutation

*TERT* (telomerase reverse transcriptase) gene mutations, particularly in the *TERT* promoter, are widely recognized as molecular markers that are closely linked to high aggressiveness and poor prognosis in many malignant tumors [40]. *TERT* mutations typically provide a molecular basis for sustained tumor growth by lengthening telomere length in tumor cells, promoting unchecked proliferation, and avoiding apoptosis. The research on *TERT* mutations in RGNTs is relatively limited, but one report indicated that one out of two cases exhibited a mutation [5]. While RGNTs are generally considered benign, the presence of *TERT* mutations in some studies suggests that these tumors may have the potential for malignant transformation. Handa et al. [5] reported a case of recurrent RGNTs harboring the *TERT C228T* mutation, which is located in the *TERT* promoter region and enhances telomerase expression, significantly extending telomere length in tumor cells. This mechanism allows tumor cells to bypass normal cellular aging processes and continue proliferating, and potentially increases the risk of recurrence and progression. The presence of the *TERT C228T* mutation may suggest that the biological behavior of RGNTs is more complex than traditionally believed, especially in recurrent or refractory cases, but its specific impact requires further investigation. Although RGNTs are generally classified as a low-grade malignancy, detecting *TERT* mutations is crucial in these cases. Such detection can help identify RGNT patients at risk of malignant transformation, allowing for a more accurate prognosis and optimized treatment strategies. In clinical practice, *TERT* mutation detection not only provides essential information for treating recurrent RGNT cases but also may help assess tumor aggressiveness and long-term survival. Therefore, detecting *TERT* mutations could become a valuable diagnostic tool in recurrent or unusually aggressive RGNT cases, facilitating the development of more personalized and targeted treatment plans.

### 5.4. IDH1/2 Gene Mutations

Mutations in the *IDH1* and *IDH2* genes are common in gliomas, particularly low-grade gliomas and secondary glioblastomas, and are usually associated with specific metabolic abnormalities [41]. Among these, *IDH1 R132H* and *IDH2 R172K* are the most prevalent mutations. These mutations alter IDH enzyme activity, leading to the production of an abnormal metabolite, 2-hydroxyglutarate (2-HG), which accumulates in cells and affects multiple epigenetic regulatory mechanisms, including changes in DNA and histone methylation, thereby driving tumor formation and progression. *IDH1* and *IDH2* mutations have been more sparsely reported in RGNTs, but their presence may indeed suggest specific biological behaviors. Solis et al. and Jayapalan et al. [6,38] confirmed the presence of *IDH1* mutations in RGNTs, although at relatively low frequencies. This finding is significant because *IDH* mutations in gliomas are typically associated with slower growth and a better prognosis. Specifically, through their metabolic effects, *IDH* mutations may confer a relatively stable and slow-growing characteristic to RGNT cells, consistent with the low-grade malignancy typically seen in RGNTs. Thus, detecting *IDH1* mutations in RGNTs could help further understand these tumors’ pathological features and may be clinically useful in differentiating RGNTs from other gliomas. For instance, *IDH* mutations are often considered positive prognostic markers in glioblastomas, indicating slower tumor growth and a better prognosis [42]. Similarly, the presence of *IDH* mutations in RGNTs may suggest a better prognosis for patients. Further studies are necessary to reveal the specific role of *IDH* mutations in RGNTs and how they affect the tumor’s biological behavior and clinical presentation. These findings expand our understanding of RGNTs and may offer new targets and strategies for future diagnosis and treatment.

### 5.5. KIAA1549-BRAF Fusion Gene

The *KIAA1549-BRAF* fusion gene is prevalent in pilocytic astrocytomas [43], but its presence in RGNTs is unclear. This fusion gene activates the MAPK signaling pathway, which may play a role in some neural tumors. Gessi et al. [44] explored the presence of the *KIAA1549-BRAF* fusion gene in RGNTs. However, despite its prevalence in pilocytic astrocytomas, the *KIAA1549-BRAF* fusion gene was not detected in the RGNT cases studied. This suggests that RGNTs may have a different molecular mechanism than pilocytic astrocytomas, a finding with significant diagnostic implications for distinguishing between the two tumors. The absence of the *KIAA1549-BRAF* fusion gene indicates that RGNTs are unique at the molecular level, possibly requiring different diagnostic and therapeutic strategies. This discovery also lays the groundwork for further exploration of RGNTs’ other molecular mechanisms, encouraging researchers to focus on other potential oncogenes and signaling pathways.

### 5.6. H3 K27M Mutation

The *H3 K27M* mutation is commonly associated with highly aggressive malignant gliomas, such as particularly diffuse midline gliomas in children [45]. The *H3 K27M* mutation, with a rate of about 14% [3,13], involves a methylation-modifying mutation in the lysine at position 27 of either the *H3F3A* or the *HIST1H3B* gene, resulting in aberrant epigenetic regulation. Such mutations are often associated with highly aggressive tumors and poor prognosis, especially in diffuse midline gliomas, usually suggesting a worsening disease course and shorter survival [46]. However, the presence of the *H3 K27M* mutation in RGNTs may lead to diagnostic confusion, as this mutation is more common in high-grade gliomas, whereas RGNTs are typically considered low-grade malignancies. Marastoni et al. [13] detected the *H3 K27M* mutation in some RGNT cases, which sparked discussions about RGNTs’ molecular characteristics and malignant potential. The study noted that although the *H3 K27M* mutation is typically linked to higher malignancy and aggressive behavior, the clinical presentation of patients with *H3 K27M*-mutated RGNTs did not match those of highly aggressive gliomas. This discrepancy suggests that clinicians should carefully interpret the significance of *H3 K27M* mutations in RGNTs. Misinterpreting this mutation could lead to the incorrect classification of a low-grade RGNT as a highly malignant tumor, potentially resulting in overtreatment with aggressive therapies that may not be appropriate for the tumor’s actual nature. Therefore, it is crucial to consider other clinical and pathological features alongside *H3 K27M* mutation status when diagnosing RGNTs. By doing so, clinicians can develop more suitable treatment plans, avoiding both overtreatment and undertreatment, and ultimately improving patient outcomes.

### 5.7. DNA Methylation Profiles

DNA methylation profiling is a key molecular biology tool widely used in tumor classification and differential diagnosis. The uniqueness of methylation profiles makes them a highly accurate means of tumor classification, revealing the origin, type, and underlying biology of tumors. Handa et al. [5] applied a DNA methylation classification tool to perform a detailed analysis of two RGNT cases in the midbrain tectum. Their results showed that RGNTs differ significantly from other known neurotumor types in terms of their methylation status, reflecting the differences in multiple genes and methylation types. The DNA methylation profile of the RGNTs showed specific methylation sites that are not usually found or sites that show a different methylation status in other CNS tumors. For instance, some key genes associated with cell proliferation and differentiation, such as PAX6, SOX10, and OLIG2, showed a high methylation status in the RGNTs. These methylation patterns are significantly different from other neural tumors like pilocytic astrocytomas (PAs), indicating the unique epigenetic regulation seen in RGNTs [47]. Furthermore, RGNTs from different anatomical locations exhibited different methylation profiles, reflecting subtype-specific methylation patterns. RGNTs in the midbrain tectum showed specific methylation patterns on genes associated with neurodevelopment (*HOXA* gene cluster), suggesting different biological behaviors in various developmental contexts. Unlike other gliomas, RGNTs’ methylation profile showed a hypomethylated or unmethylated state on certain tumor suppressor genes (*TP53* and *RB1*), which are typically hypermethylated in diffuse gliomas and other highly malignant tumors [47]. This hypomethylated state aligns with RGNTs’ relatively benign biological behavior, further supporting its classification as a distinct pathological entity. Through more in-depth DNA methylation profiling, researchers can better understand RGNTs’ biological behavior and provide reliable molecular signatures for developing individualized treatment plans. These specific methylation profiles not only help more accurately identify and classify RGNTs but also reveal their essential differences from other CNS tumors at the molecular level, promoting the development of more precise diagnostic and therapeutic approaches for RGNTs.

## 6. The Immune Microenvironment of RGNTs

The immune microenvironment of RGNTs is markedly heterogeneous, with differences observed across tumor subtypes and locations [39]. The immune microenvironment of RGNTs can be categorized into “cold” and “hot” types based on the level of immune cell infiltration and the activity of immune responses within the tumor microenvironment. In “hot” RGNTs, there is significant infiltration of peripheral immune cells, including large numbers of T-lymphocytes, monocytes, and neutrophils, which correlates with heightened immune response activity in the tumor. “Hot” RGNTs are typically associated with higher immunoreactivity, showing abundant immune cell stromal characteristics, which may suggest a more malignant biological behavior. In contrast, “cold” RGNTs exhibit less immune cell infiltration, primarily involving CNS-resident immune cells like microglia. These “cold” tumors display lower immune reactivity, greater immune evasion, and slower tumor progression. Notably, in “hot” RGNTs, the expression of certain chemokines, such as CCL18 and CXCL6, is significantly upregulated. These chemokines play a crucial role in mobilizing and directing immune cells to infiltrate the tumor microenvironment, potentially promoting tumor progression.

Studies also indicate that the immune microenvironment of RGNTs is related to the specific anatomical location of the tumor, with significant differences in immune cell infiltration and activation observed across different sites. RGNTs in the midbrain tectum tend to have higher levels of immune infiltration and more active immune responses, while RGNTs in other regions may exhibit a more subdued immune state. These findings offer new insights into the biological behavior of RGNTs, their mechanisms of immune evasion, and their impact on therapeutic responses. The immune microenvironment of RGNTs is complex and diverse, and this diversity not only plays a critical role in tumor maintenance and progression but also provides a crucial foundation for personalized immunotherapeutic strategies. Understanding and targeting these immune microenvironment features will be an important direction for future RGNT treatment.

## 7. Malignant Transformation of RGNTs

Although RGNTs generally exhibit low malignancy, their potential for malignant transformation should not be underestimated. Kwon detailed a case of spontaneous malignant transformation of an RGNT into a glioblastoma (GBM) seven years after a complete resection [48]. This finding was the first to reveal that RGNTs have the potential for malignant transformation over long-term follow-up, indicating that even when initially diagnosed as benign, these tumors may progress to more aggressive forms. Kwon et al. explored the biphasic organizational structure of RGNTs, characterized by the presence of both neuronal and glial components, with each playing distinct roles in the tumor’s biological behavior. During the malignant transformation into glioblastoma, this biphasic structure undergoes significant changes, with the original neuronal component gradually diminishing or becoming insignificant, while the glial component, particularly the highly proliferative glial cells, becomes more dominant. This shift implies that the tumor progressively loses its typical RGNT histological features and begins to exhibit the highly heterogeneous and aggressive cellular characteristics of glioblastoma. These changes were closely associated with a significant increase in the Ki-67 proliferation index to 20%, reflecting the profound biological alterations that occurred during the malignant transformation.

In the transformed tumors, mutations in the *TP53* gene were observed, which is a common molecular change in many highly malignant tumors. This mutation is closely linked to cell cycle deregulation and enhanced tumor aggressiveness. Conversely, the study did not detect mutations in the *IDH1/2* and *BRAF* genes in the transformed tumors, which are typically found in other gliomas. Additionally, the *MGMT* promoter was not methylated, which is consistent with the highly aggressive and treatment-resistant nature of the transformed tumors. These combined molecular characteristics suggest that although RGNTs initially present with low malignancy, they still have the potential to progress into a highly aggressive tumor under certain conditions. The significance of this case report lies in its reminder to clinicians that even RGNTs classified as WHO grade I require long-term follow-up to prevent a potential malignant transformation. This finding not only provides important guidance for clinical management but also highlights the need for further research into the molecular mechanisms of RGNTs to better understand their biological behavior and optimize therapeutic strategies.

## 8. Diagnostic Challenges and Misdiagnosis

The diagnosis of RGNTs is complex, particularly when accompanied by other common mutations such as *H3 K27M*, which may lead to misdiagnosis as other types of gliomas. The complexity of these molecular features highlights the importance of accurate diagnosis and the necessity of considering multiple molecular markers when developing treatment plans. Yang et al. [3] provided a comprehensive description of the histological, molecular, and clinical features of RGNTs, emphasizing its differential diagnosis from other CNS tumors. Although RGNTs are usually benign, their complex molecular characteristics can be confused with those of other malignant tumors, necessitating a combined assessment of multiple molecular markers for an accurate diagnosis. Marastoni et al. [13] specifically discussed the misleading nature of the *H3 K27M* mutation in RGNTs, pointing out that while this mutation is common in more aggressive gliomas, its presence in RGNTs can lead to misdiagnosis. Therefore, an accurate molecular diagnosis is crucial to avoid unnecessary overtreatment.

## 9. Treatment Strategies

Surgical resection remains the primary treatment for RGNTs, with a gross total resection (GTR) being considered the optimal treatment due to its association with a better prognosis. Michel et al. found that patients who underwent a total resection had lower recurrence rates and better long-term outcomes, emphasizing the necessity of long-term postoperative MRI follow-up to detect potential tumor recurrences and malignant transformations [23]. In cases where a complete resection is not possible or in the event of recurrence, Gamma Knife radiosurgery (GKR) has shown effectiveness and is recommended as an adjunct therapy [24].

Pharmacological treatment strategies for RGNTs remain an area of ongoing research, with increasing attention towards precision medicine. While there is no conclusive evidence supporting the use of bromocriptine for treating RGNTs specifically, some reports suggest its efficacy in mitigating postoperative cerebellar mutism, a condition often associated with posterior fossa surgery [49]. However, recent advances in molecular biology have highlighted several drug-targetable mutations in RGNTs, offering potential therapeutic avenues, particularly in the context of personalized medicine and targeted therapies.

*FGFR1* mutations are among the most prevalent genetic alterations identified in RGNTs, particularly within the tyrosine kinase domain. The FGFR1 signaling pathway plays a critical role in regulating cellular processes such as proliferation, migration, and differentiation, making it an ideal target for cancer therapy. Erdafitinib (approved for metastatic bladder cancer) and Pemigatinib (approved for cholangiocarcinoma) are FGFR inhibitors that have demonstrated clinical efficacy in treating tumors harboring FGFR mutations [50]. By inhibiting aberrant FGFR activation, these agents effectively prevent uncontrolled cellular proliferation. Given the frequent presence of FGFR1 mutations in RGNTs, these inhibitors represent promising candidates for targeted therapy in this tumor subtype. Nevertheless, additional clinical evidence specific to RGNTs is required to substantiate their efficacy in this context.

Similarly, *PIK3CA* mutations, another key driver in RGNTs pathogenesis, promote tumor growth and survival through activation of the PI3K/AKT/mTOR signaling axis. Targeting this pathway is a well-established strategy in oncology. Alpelisib, an FDA-approved PI3K inhibitor for advanced breast cancer with *PIK3CA* mutations, has been shown to significantly extend progression-free survival and improve overall survival [51]. While no clinical trials have yet been conducted specifically for RGNTs with *PIK3CA* mutations, insights from solid-tumor studies may inform future therapeutic approaches for RGNTs.

*TERT* promoter mutations contribute to tumorigenesis by enhancing telomere maintenance, enabling continuous cell proliferation and survival. *TERT* is a recognized target in multiple cancer types. Imetelstat, a telomerase inhibitor, has undergone clinical trials in patients with CNS tumors, including recurrent medulloblastoma, high-grade glioma (HGG), and ependymoma. In these trials, imetelstat effectively inhibited telomerase activity in both tumor tissue and peripheral blood mononuclear cells (PBMCs). However, the trials were prematurely halted due to severe hematological toxicity, particularly thrombocytopenia [52]. The applicability of imetelstat for RGNTs harboring *TERT* mutations remains to be explored, warranting further investigation in future studies.

*IDH1* mutations, although infrequent in RGNTs, are more commonly observed in gliomas, where they drive tumorigenesis through epigenetic dysregulation. *IDH* inhibitors have shown efficacy in gliomas with *IDH1* mutations and hold potential for application in RGNTs [41], albeit evidence for their use in this tumor subtype remains limited.

Furthermore, aberrant DNA methylation is another molecular feature that may be targeted in RGNTs. Drugs such as azacitidine and decitabine, both of which are demethylating agents, have been successfully utilized in the treatment of hematologic malignancies [53]. These agents function by reactivating silenced tumor suppressor genes, thereby inhibiting tumor progression. Studies have suggested that the DNA methylation profile of RGNTs may differ significantly from that of other CNS tumors, presenting a unique epigenetic signature that could be exploited for therapeutic intervention [8]. In particular, highly aggressive RGNT cases may benefit from the modulation of DNA methylation as a potential therapeutic approach.

While the rarity of RGNTs poses challenges for conducting large-scale clinical trials, the advent of precision medicine and targeted therapies directed at specific genetic alterations offers a promising future for patients with recurrent or refractory RGNTs. Further research and clinical trials are essential to validate these approaches and integrate them into standard treatment protocols.

## 10. Directions for Future Research

Although the key molecular features of RGNTs have been identified, their detailed molecular mechanisms still require further exploration. Future research should integrate genomic, transcriptomic, proteomic, and epigenetic data to fully decode the molecular pathology of RGNTs. Additionally, investigating the clinical applications of these molecular features, such as targeted therapies, will be a significant focus going forward. Precision in molecular diagnosis and treatment strategies will be especially important in managing cases with a recurrence risk or potential for malignant transformation.

## 11. Conclusions

RGNTs have become an important area of study in neuro-oncology due to their unique histological features and complex molecular mechanisms. Advances in molecular biology have deepened our understanding of the molecular characteristics and pathological mechanisms of RGNTs, opening new possibilities for individualized treatment in the future. By employing precise molecular diagnostics and multidisciplinary treatment strategies, we can expect to improve the prognosis of RGNT patients and advance research and clinical practices in this field.

## Data Availability

No new data were created or analyzed in this study.
